# Improved drought tolerance in wheat plants overexpressing a synthetic bacterial cold shock protein gene *SeCspA*

**DOI:** 10.1038/srep44050

**Published:** 2017-03-10

**Authors:** Tai-Fei Yu, Zhao-Shi Xu, Jin-Kao Guo, Yan-Xia Wang, Brian Abernathy, Jin-Dong Fu, Xiao Chen, Yong-Bin Zhou, Ming Chen, Xing-Guo Ye, You-Zhi Ma

**Affiliations:** 1 Institute of Crop Science, Chinese Academy of Agricultural Sciences (CAAS)/National Key Facility for Crop Gene Resources and Genetic Improvement, Key Laboratory of Biology and Genetic Improvement of Triticeae Crops, Ministry of Agriculture, Beijing 100081, China; 2 Shijiazhuang Academy of Agricultural and Forestry Sciences, Research Center of Wheat Engineering Technology of Hebei, Shijiazhuang, Hebei 050041, China; 3 Center for Applied Genetic Technologies, Department of Plant Sciences, University of Georgia, 30602, Athens, GA, United States

## Abstract

Cold shock proteins (CSPs) enhance acclimatization of bacteria to adverse environmental circumstances. The *Escherichia coli* CSP genes *CspA* and *CspB* were modified to plant-preferred codon sequences and named as *SeCspA* and *SeCspB*. Overexpression of exogenous *SeCspA* and *SeCspB* in transgenic *Arabidopsis* lines increased germination rates, survival rates, and increased primary root length compared to control plants under drought and salt stress. Investigation of several stress-related parameters in *SeCspA* and *SeCspB* transgenic wheat lines indicated that these lines possessed stress tolerance characteristics, including lower malondialdehyde (MDA) content, lower water loss rates, lower relative Na^+^ content, and higher chlorophyll content and proline content than the control wheat plants under drought and salt stresses. RNA-seq and qRT-PCR expression analysis showed that overexpression of *SeCsp* could enhance the expression of stress-responsive genes. The field experiments showed that the *SeCspA* transgenic wheat lines had great increases in the 1000-grain weight and grain yield compared to the control genotype under drought stress conditions. Significant differences in the stress indices revealed that the *SeCspA* transgenic wheat lines possessed significant and stable improvements in drought tolerance over the control plants. No such improvement was observed for the *SeCspB* transgenic lines under field conditions. Our results indicated that *SeCspA* conferred drought tolerance and improved physiological traits in wheat plants.

Abiotic stresses such as drought, salt, heat, and cold are major environmental factors that affect plant growth and development^1^,^2^. Increasing numbers of genetically modified plants that have the ability to resist abiotic stresses have been reported recently^3^,^4^,^5^. Extensive field evaluations have demonstrated that the down-regulation of the ethylene biosynthetic pathway can improve grain yield in maize under drought conditions^6^. Overexpression of a C4 photosynthesis enzyme enhanced transgenic rice tolerance to drought stress^7^. Coexpression of ABA-Insensitive3 (ABI3)/Viviparous1 and an AtABI5 transcription factor in cotton enhanced drought stress adaptation under limited irrigation conditions^8^. Some studies showed that fungal and bacterial genes can be used to confer resistance to abiotic stresses in plants. For example, tobacco plants exhibited dehydration and necrosis under severe water-deficit conditions; but these symptoms were less severe in transgenic tobacco plants with the yeast trehalose-6-phosphate synthase gene^9^. Maize plants are sensitive to water-deficit stress throughout the growing season, but water deficit during the vegetative growth phase is of particular concern, as it typically leads to reduced grain yields. Bacterial cold shock protein (CSP) genes enhanced resistance to drought stress and improved grain yield in maize under drought conditions^10^.

CSPs are essential for assisting bacterial growth under low temperature conditions and in enhancing bacterial acclimatization to low temperatures^11^. The *CspA* and *CspB* genes play major roles in bacterial responses to low temperature^12^. The effect of temperature fluctuations on *CspA* and *CspB* has been experimentally defined^13^. During temperature cycling, the ratios of CspA or CspB to total protein change as temperatures vary^14^; transcription of both genes increases at low temperatures and decreases with increasing temperatures. CspA and CspB are thought to enhance protein translation at low temperatures through the elimination of stabilized RNA secondary structures^15^,^16^.

Water-deficit stress has become a serious problem in global agriculture and severely affects the growth of crops; it is of particular concern in northern and northwestern China, where the main winter wheat production areas are located^17^. In these areas, more than 70% of annual precipitation falls from June to September, whereas precipitation meets only 20 to 30% of the water requirement for winter wheat during the winter wheat growing season from February to the middle of June^18^. Therefore, the discovery of genetic sources of drought tolerance has become an urgent priority in wheat improvement efforts. The study reported here was conducted with field experiments and showed that the 1000-grain weight and the grain yield of wheat was significantly increased in transgenic wheat expressing *SeCspA* under drought conditions, resulting in improved wheat drought tolerance.

## Results

### Overexpression of *SeCspA* and *SeCspB* increased germination rates in *Arabidopsis* under abiotic stresses

We transformed *Arabidopsis* with *SeCsp (SeCspA* and *SeCspB*) and obtained four new *35 S:SeCsp* transgenic lines. The expression levels of *SeCsp* in 4 lines are shown in Fig. S2. One hundred homozygous T3 seeds of the *35 S::SeCspA* and *35 S::SeCspB* transgenic lines, and control plants, were used for germination. The result showed, under normal growth conditions, the seed germination rates of the *35 S::SeCspA* and *35 S::SeCspB* transgenic lines and control plants showed no obvious differences, and exceeded 95%. With increasing NaCl concentrations, the germination rates of the *35 S::SeCspA* and *35 S::SeCspB* transgenic *Arabidopsis* lines were significantly higher than that of the control plants, although the germination of both the transgenic lines and the control plants showed a declining trend as the NaCl concentrations increased (Fig. S3). In media supplemented with 150 mM NaCl, the *35 S::SeCspA* and *35 S::SeCspB* transgenic *Arabidopsis* lines germinated at 70% and 56%, respectively, as compared with about 33% germination for the control plants.

To analyze the effect of polyethylene glycol (PEG 6000)-simulated drought stress on the germination of the *35 S::SeCspA* and *35 S::SeCspB* lines, seeds were placed on half-strength solid MS medium supplemented with various concentrations of PEG. After one week, the germination rates of the *35 S::SeCspA* and *35 S::SeCspB* transgenic lines were significantly higher than those of control plants under PEG stress at all PEG concentrations (Fig. S4). For example, the germination rates of the *35 S::SeCspA* and *35 S::SeCspB* transgenic lines were more than 62.3% and 56.3% under 8% PEG, respectively, as compared to the 37.5% rate of the control seeds (Fig. S4B,C). These results indicated that overexpression of *SeCspA* and *SeCspB* enhanced salt and PEG tolerance of the transgenic plants at the germination stage.

### Tolerance of transgenic *Arabidopsis* against salt, cold and drought stresses

**Figure 1:**
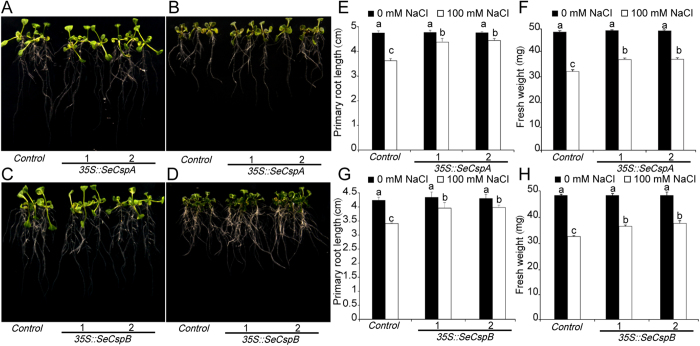
Responses of transgenic *Arabidopsis* lines and control plants under salt stress. (**A**) and (**C**) Phenotypes of the *SeCspA* and *SeCspB* transgenic *Arabidopsis* lines grown on normal MS agar medium. (**B**) and (**D**) Phenotypes of the *SeCspA* and *SeCspB* transgenic *Arabidopsis* lines grown on high-salt medium (100 mM NaCl). (**E**) and (**G**) Primary root lengths of the *Arabidopsis* plants. (**F**) and (**H**) Fresh weights of the aerial parts of *Arabidopsis* plants. Three biological replicates were averaged and statistically analyzed using Duncan’s test. Vertical bars bearing different letters in (**E**), (**F**), (**G**) and (**H**) indicate significant differences at P < 0.05. Bars indicate the standard error of the mean (SE).

To investigate whether or not transformation of plants with the *35 S::SeCspA* and *35 S::SeCspB* constructs affected salt tolerance during plant growth, 9-day-old *Arabidopsis* seedlings were transferred to MS plates supplemented with 100 mM NaCl. In the absence of NaCl, there were no significant differences in growth characteristics between the transgenic lines and control plants (Fig. 1A,C). When grown in the presence of 100 mM NaCl for one week, some leaves of the control plants began to yellow, whereas those of the two *35 S::SeCspA* transgenic lines remained green (Fig. 1B), and the primary root lengths of control plants were 23.5% shorter than the untreated controls, whereas the two *35 S::SeCspA* transgenic lines showed reductions of 8.1 and 6.5%. Salt stress reduced the fresh weights of control plants by 33.0%, compared to 23.7% to 23.5% reductions for the two *35 S::SeCspA* transgenic lines (Fig. 1E,F). Similar phenotypic results were observed for the *35 S::SeCspB* transgenic plants. After salt treatment for one week the two *35 S::SeCspB* transgenic lines had significantly longer primary root lengths and significantly higher fresh weights than did control plants (Fig. 1D,G and H). For example, under salt treatment, the two *35 S::SeCspB* transgenic lines showed fresh weight reductions of 2.9 and 3.8% in comparison with normal conditions, compared to a corresponding reduction of 10.1% for control plants (Fig. 1H).

**Figure 2:**
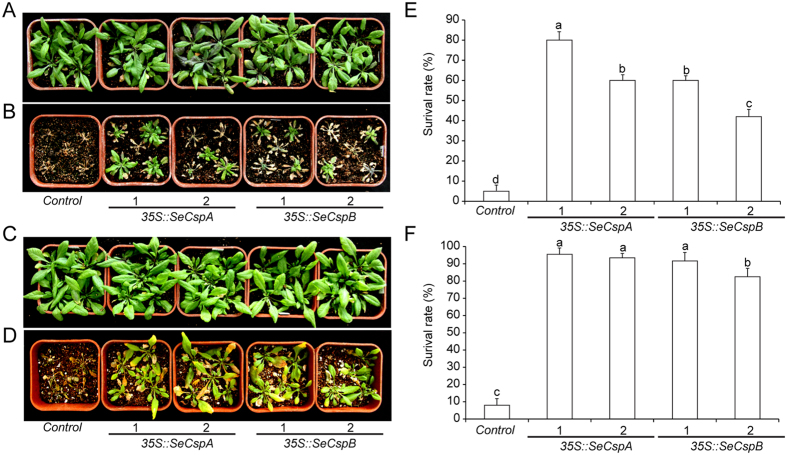
Phenotypes of transgenic *Arabidopsis* lines and control plants subjected to stress treatments. (**A**) and (**C**) Phenotypes of transgenic *Arabidopsis* lines under normal condition. (**B**) Phenotypes after cold stress treatment. Plants were photographed after one week of recovery under normal conditions. (**D**) Phenotypes of transgenic *Arabidopsis* lines grown under dehydration conditions for one week. Photographs were taken after one week of recovery under normal conditions. (**E**) and (**F**) Survival rates of *Arabidopsis* lines. Vertical bars bearing different letters in (**E**) and (**F**) indicate significant differences at P < 0.05 and error bars represent standard errors.

To investigate the tolerance of the transgenic *Arabidopsis* lines to cold stress, three-week-old *Arabidopsis* seedlings grown under normal conditions were transferred to −5 °C for 12 h for cold treatment and then returned to normal growth conditions for recovery. During the recovery phase, the transgenic *Arabidopsis* lines grew well and only a few leaves underwent yellowing, whereas the leaves of the control plants showed severe wilting and curling. After one week of recovery week, more than 40% of the *35 S::SeCspA* and *35 S::SeCspB* transgenic plants survived compared to only 5% survival for the control plants (Fig. 2B,E).

To explore the drought tolerance of the transgenic *Arabidopsis* lines, three-week-old seedlings were exposed to water deficit stress for one week, and then rewatered in a recovery period of one week (Fig. 2D). As shown in Fig. 2F, after one week of recovery, more than 92% and 80%, respectively, of the *35 S::SeCspA* and *35 S::SeCspB* transgenic *Arabidopsis* plants had survived, compared to only 8% of the control plants. These results suggested that overexpression of *SeCspA* and *SeCspB* appeared to increase salt, cold, and drought tolerance in transgenic *Arabidopsis* plants.

### The effect of salt stress on the transgenic wheat lines

**Figure 3:**
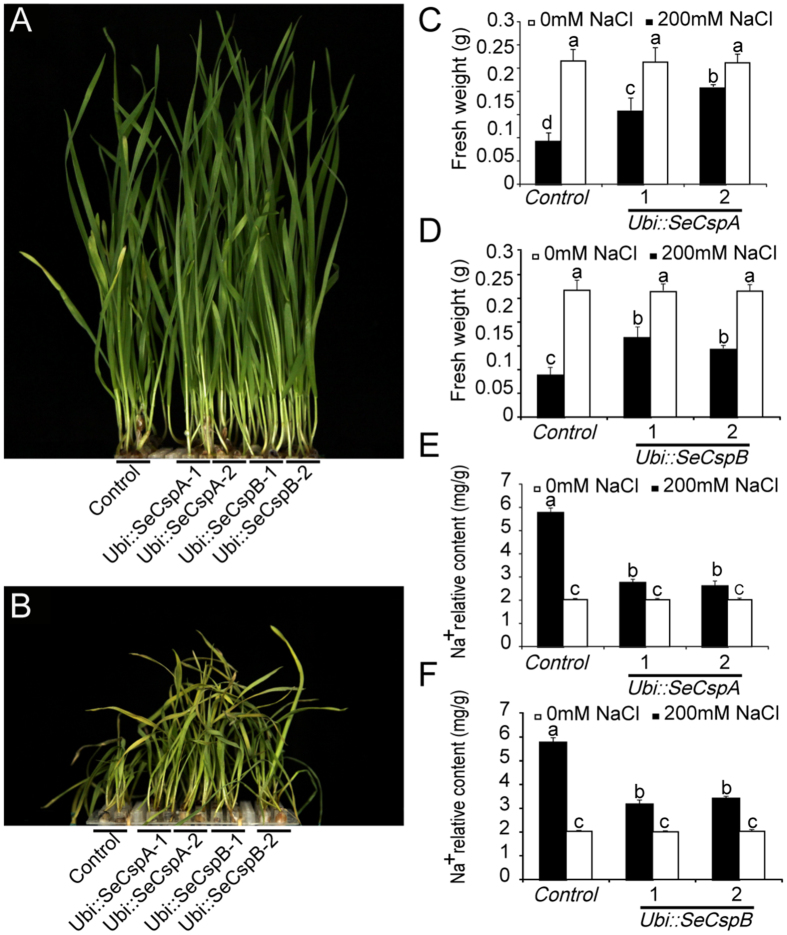
Responses of the transgenic lines and parental wheat line (control) under salt stress. (**A**) Phenotypes of transgenic wheat lines grown under normal conditions for two weeks. (**B**) Phenotypes of transgenic wheat lines grown under high-salt (200 mM NaCl) stress. (**C**) and (**D**) Fresh weights of the transgenic wheat lines and control wheat lines. Measurements were made after 7-day-old plants were treated with NaCl for one week. (**E**) and (**F**) Relative Na^+^ content. Measurements were made after 7-day-old plants were treated with NaCl for 6 h. Vertical bars bearing different letters in (**C**,**D**,**E**), and (**F**) indicate significant differences at P < 0.05 and error bars represent standard errors.

*Ubi::SeCspA* and *Ubi::SeCspB* transgenic wheat lines were subjected to high-salt stress (200 mM NaCl) (Fig. 3). As expected, control plants began to wilt after one week of salt stress, whereas the *Ubi::SeCspA* and *Ubi::SeCspB* transgenic lines continued to thrive (data not shown). After two weeks, the control plants had completely wilting, but the transgenic lines, especially the two *Ubi::SeCspA* lines, remained upright (Fig. 3B). The fresh weight measurement data showed that in the absence of salt treatment, there were no significant differences among the transgenic and control wheat plants. Salt stress reduced the fresh weights of control plants by 64.3%, compared to 19.4% to 39.8% reductions for the two *Ubi::SeCspA* transgenic lines and 31.9% to 41.2% reductions for the two *Ubi::SeCspB* transgenic lines (Fig. 3C,D).

To further analyze the mechanism underlying the observed salt-tolerance phenotype, the relative Na^+^ content of the transgenic and control wheat plants was measured using an atomic absorption spectrometer. The results showed that the Na^+^ content of the control wheat plant and all of the *Ubi::SeCspA* and *Ubi::SeCspB* transgenic wheat lines was significantly increased after the plants were subjected to salt stress (200 mmol/L NaCl) for 6 h. However, the Na^+^ content of the transgenic wheat plants increased less dramatically than that of the control wheat plants (Fig. 3E,F). This result suggests that the transgenic wheat plants exhibited a decreased Na^+^ content into the cells under salt stress, but the control wheat plant presented a higher Na^+^ content into the cells due to their high membrane permeability.

### The phenotypes of transgenic wheat lines were not affected by low temperature

To investigate potential plant responses to low temperature, 7-day-old transgenic and control wheat seedlings were transferred to −4 °C conditions for one week. As expected, the low temperature inhibited plant growth. There were no significant differences in the above-ground growth or root length among the transgenic lines and the control plants (Fig. S7E,F).

To further analyze whether transgenic wheat lines were affected by low temperature, the relative electric conductivity experiment was carried out. The result showed, under normal conditions (25 °C), there were no significant differences in the relative electric conductivity among the transgenic lines and the control plants. After −8 °C treatment for 2 h, although the relative electric conductivity of transgenic and control wheat seedlings leaves had a great increase, there were still no differences among the transgenic lines and the control plants (Fig. S7G,H).

### Survival rates and effects on physiological indices of transgenic wheat lines under drought stress

**Figure 4:**
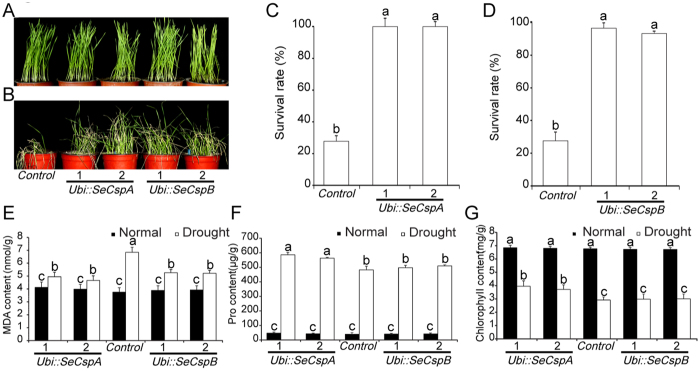
Phenotypes of transgenic wheat lines and parental wheat plants (control) under drought stress. (**A**) Phenotypes of transgenic wheat lines grown under normal conditions for three weeks. (**B**) Phenotypes of the transgenic wheat lines after rehydration for one week. (**C**) and (**D**) Survival rates of the transgenic wheat lines. (**E**) MDA content. (**F**) Free proline content. (**G**) Chlorophyll content. Measurements were made after 7-day-old plants were treated with drought conditions for one week. Vertical bars bearing different letters in (**C**,**D**,**E**,**F**), and (**G**) indicate significant differences between transgenic and control wheat plants at P < 0.05 and error bars represent standard errors.

To test whether or not the *Ubi::SeCspA* and *Ubi::SeCspB* constructs conferred improved drought resistance to wheat plants, 7-day-old seedlings were exposed to severe water deficit. After one week, the control plants exhibited serious wilting and death; the symptoms of the *Ubi::SeCspA* and *Ubi::SeCspB* transgenic lines were considerably less severe (Fig. 4B). After re-watering for one week, 97.6% and 93.3%, respectively, of the *Ubi::SeCspA* and *Ubi::SeCspB* transgenic plants recovered and grew normally, while only 29.2% of the control plants survived and grew normally (Fig. 4C,D).

After 7 days of drought treatment, the malondialdehyde (MDA) content of the control plants increased rapidly and reached 6.85 nmol/g, an increase of 82.7% compared with growth under the normal irrigation condition. Drought treatment increased the MDA content of the *Ubi::SeCspA* and *Ubi::SeCspB* transgenic wheat lines by only 16.6%, 20.1%, 33.3%, and 35.7%, respectively, relative to their counterparts grown under normal irrigation conditions (Fig. 4E). Thus, the MDA content remained relatively stable in the transgenic wheat lines compared to the control plants.

Proline, an osmoprotectant that is known to promote drought resistance, accumulates in plant cells during drought stress^19^. When subjected to severe drought stress, significant increases in proline content were detected in both the *Ubi::SeCspA* and *Ubi::SeCspB* transgenic lines and in the control wheat plants (Fig. 4F). The two *Ubi::SeCspA* transgenic lines showed the highest proline content, reaching 563 and 586 μg/g under drought stress conditions, whereas the control wheat reached 482 μg/g. Although there were increases, relative to the control plants, in the proline content (497 and 510 μg/g) of the two *Ubi::SeCspB* transgenic lines, the differences were not statistically significant (Fig. 4F).

It is known that chlorophyll content directly affects photosynthetic efficiency^20^. Under normal conditions, there were no significant differences in chlorophyll content among the transgenic wheat lines and control plants (Fig. 4G). Drought stress decreased the chlorophyll content in the transgenic lines and in the control plants, relative to normal irrigation conditions. Under drought stress, the transgenic lines had less severe reductions in chlorophyll content (43.8 to 45.7%) than did the control plants (57.1%), but only the differences for the *Ubi::SeCspA* transgenic lines were statistically significant (Fig. 4G). The results of MDA content, free proline content and chlorophyll content revealed that the two *Ubi::SeCspA* transgenic lines had relatively stronger drought resistance than the control plants under water-deficit conditions.

### Effect of ABA on stomatal movement

**Figure 5:**
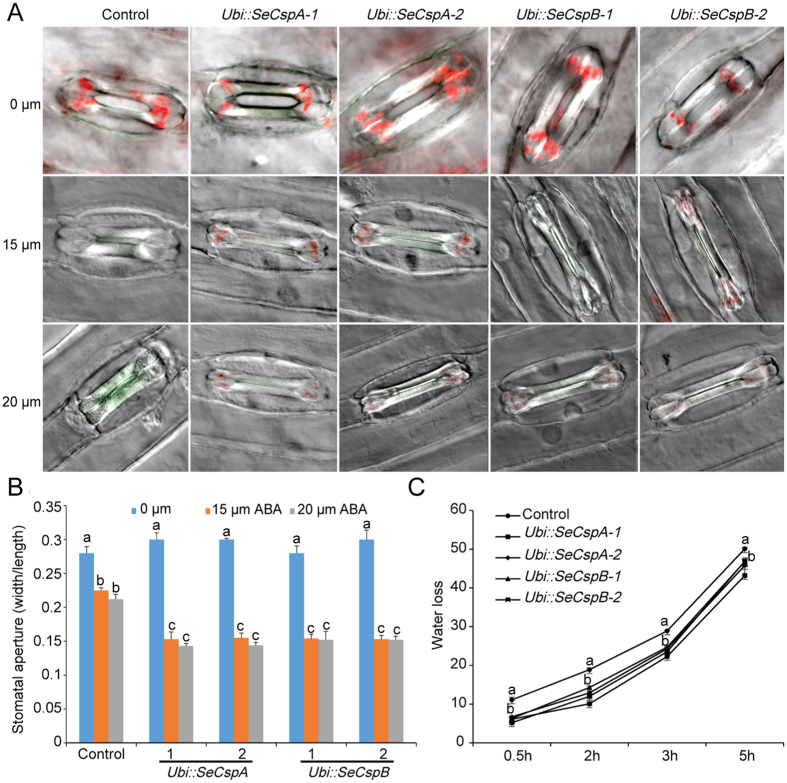
Measurements of stomatal closure and water loss in transgenic lines and control plants. (**A**) and (**B**) Effects of ABA-induced stomatal closure. (**C**) Water loss from transgenic lines and control plants. Vertical bars bearing different letters in (**B**) indicate significant differences between transgenic and control wheat plants at P < 0.05. Different letters in (**C**) indicate in one treatment significant differences between transgenic and control wheat plants at P < 0.05. Error bars represent standard errors.

ABA-mediated stomatal movement was monitored in the transgenic lines and the control plants. Seven-day-old wheat leaves were treated with ABA for 2 h, and stomatal aperture was calculated as the ratio of width to length. The mean stomatal apertures of the control and transgenic wheat leaves decreased concurrently as the concentrations of ABA increased (Fig. 5A). However, the guard cells of the transgenic lines exhibited greater sensitivity to ABA-induced stomatal closure than did those of the control leaves (Fig. 5B). The stomata of the transgenic wheat leaves closed relatively more rapidly than did the control stomata following treatment with exogenous ABA.

Further, we also compared the rate of water loss of detached leaves during dehydration between control and transgenic lines plants. The transgenic wheat line leaves showed lower rates of water loss than the control wheat plant leaves during dehydration (Fig. 5C). For example, at the 0.5 h time point of the dehydration treatment, the control wheat plant leaves revealed a water loss of 10.1% compared to 6.1–6.7% for the two *Ubi::SeCspA* transgenic lines, and 5.2–6.3% for the two *Ubi::SeCspB* transgenic lines. At the 2 h time point of the dehydration experiment, the water loss rates of the two *Ubi::SeCspA* transgenic line leaves were less than 10.1% and 12.9% and the two *Ubi::SeCspB* transgenic line leaves were less than 12% and 14.3%, respectively, compared to 19.2% for the control plant leaves. This result was consistent with the stomatal movement from transgenic wheat plants after drought stress, suggesting that detached leaves from the transgenic lines showed higher ability to withstand tissue dehydration.

### Analysis of SeCSP enhanced the expression of stress-responsive genes

To better understand the differential expression of stress-related genes between the controls and transgenic wheat, an RNA sequencing (RNA-seq) approach was used. The raw sequence reads were deposited into the Ensembl Plants and the National Center for Biotechnology Information SRA database. The assembled transcripts that were differentially expressed in transgenic wheat plants are listed in Table S1.

In the context of the influence of abiotic stress by *SeCspA* and *SeCspB*, we focused on the transcripts that were differentially expressed between the controls and transgenic wheat. Based on their annotation, we selected transcripts that were upregulated 2-fold in *SeCspA* and *SeCspB* transgenic wheat plants compared with the control plants (Table S1). According to their functions, the corresponding genes are divided into three categories which are stress response-related genes (e.g., *WD40, WRKY, LEA* and *GST*), signal transduction-related genes (e.g., receptor kinase) and energy metabolism-related genes (e.g., *Plasma membrane ATPase* and *Beta-glucosidase*) (Table S1). These proteins have previously been reported to act either directly or indirectly in abiotic stress responses.

**Figure 6:**
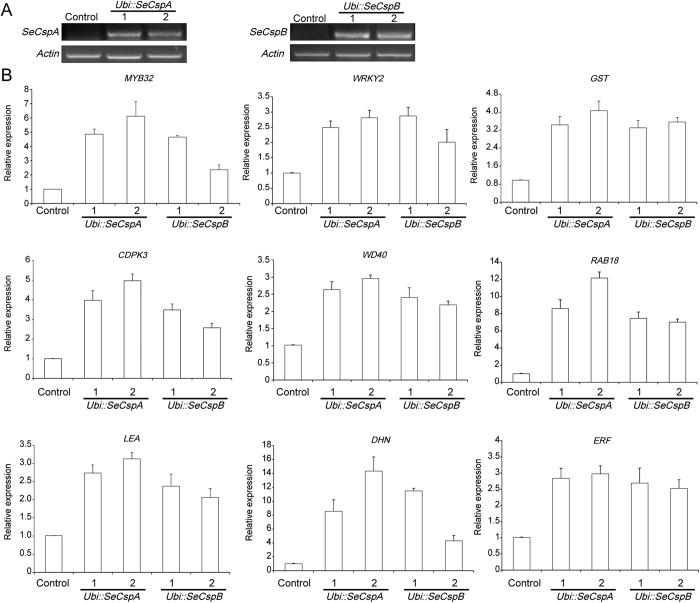
Variation in the transcript levels of stress-related genes in transgenic and control wheat plants. (**A**) Transcript levels of *SeCspA* and *SeCspB* in transgenic wheat lines. (**B**) The transcript levels of stress-related genes in transgenic wheat lines.

To validate the data from the RNA-seq digital expression analysis, we performed qRT-PCR assays of nine abiotic response-related genes, which investigated encode Calcium-dependent protein kinase (*TaCDPK3*), MYB transcriptional factor protein (*TaMYB32*), WD40 transcriptional factor protein (*TaWD40*), WRKY transcriptional factor protein (*TaWRKY2*), AP2-like ethylene-responsive transcription factor protein (*TaERF3*), ABA-responsive protein (*TaRAB18*), Late-embryogenesis abundant proteins (*TaLEA*), Glutathione S-transferase protein (*GST*) and a dehydrin protein (*TaDHN*) (Fig. 6). These genes are known to be involved in drought, salt, and ABA responses, and each of them had more than 2-fold higher expression in transgenic plants than in the control wheat plants (Fig. 6B). Although there were some anomalous quantitative differences, the trends of gene expression changes detected by the two different approaches were generally consistent (Fig. 6), thereby confirming the validity of the RNA-seq data. These results indicated that the introduction of exogenous *Ubi::SeCspA* and *Ubi::SeCspB* resulted in increased expression of some stress response genes.

### Drought resistance of transgenic wheat lines in the field

**Figure 7:**
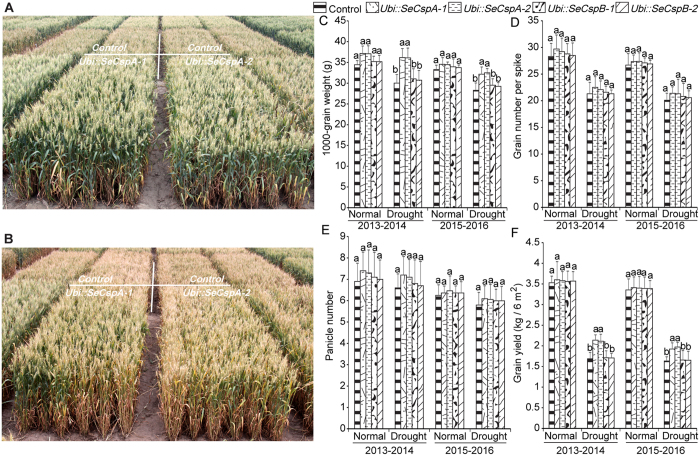
Phenotypes of transgenic lines and the control plants in May 2013 and 2016. (**A**) Transgenic wheat lines grown under normal conditions. (**B**) Transgenic wheat lines grown under drought conditions. (**C**) 1000-grain weight. (**D**) Grain number per spike. (**E**) Panicle number. (**F**) Grain yield. Vertical bars bearing different letters in (**C**,**D**,**E**) and (**F**) indicate in one treatment significant differences between transgenic and control wheat plants at P < 0.05 and error bars represent standard errors.

When grown under normal field conditions, the transgenic wheat lines and the control plants similar growth characteristics (Fig. 7A). At harvest, some agronomic traits of the transgenic and control wheat plants were analyzed. Panicle number, 1000-grain weight, and grain number per spike are important components of yield. Under normal irrigation conditions, despite small increases in the 1000-grain weight and the grain yield of the transgenic lines, The Duncan’s test showed no significant differences in agronomic traits among the transgenic lines and the control plants (Fig. 7C,F). Under drought stress, the 1000-grain weight of the two *Ubi::SeCspA* transgenic lines increased by 19.2 and 20.2% (average of two years), compared to the control plants (Fig. 7C). The grain yield of the two *Ubi::SeCspA* transgenic lines exceeded that of the control plants by 24.1 and 24.5% (average of two years) (Fig. 7F). Although there were increases, relative to the control plants, in the 1000-grain weight (1.5 and 2.0%) (average of two years) and the grain yield (1.3 and 2.1%) (average of two years) in the two *Ubi::SeCspB* transgenic lines, the differences were not statistically significant (Fig. 7C,F). We also investigated plant height and heading time, but no differences were found between the transgenic lines and control plants in Tables S2 and S3.

**Figure 8:**
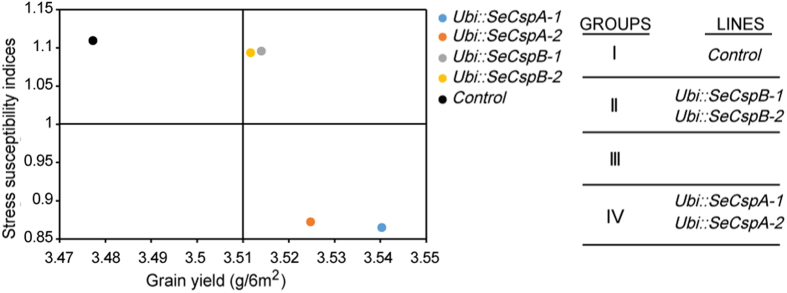
Cluster analysis of grain yield (plants grown under normal conditions) and drought susceptibility index (average of two years).

The stress tolerance index (*STI*) and stress tolerance (*TOL*) are two measures used to identify cultivars that produce high yields under both normal and stress conditions. When drought stress is severe, the stress susceptibility index (*SSI*) is thought to be a more useful index for evaluating drought tolerant cultivars^21^. Correlation analysis of the present data in Table S4 suggested that the *SSI* and *TOL* of each wheat variety were negatively correlated with the different traits under both normal and drought stress conditions, therefore, as shown in Table 1, the greater the *TOL* and *SSI* values, the more severe the reduction in yield in drought stress conditions, and the higher drought sensitivity. In order to better understand drought resistance of each wheat variety, we analyzed *SSI* and grain yield (under normal conditions) by cluster analysis, and found that the transgenic wheat lines and the control plants clustered into four groups: group I, low yield with the low drought resistance (control wheat plants); group II, high yield with the low drought resistance (*Ubi::SeCspB-1* and *Ubi::SeCspB-2*); group IV, high yield with the high drought resistance (*Ubi::SeCspA-1* and *Ubi::SeCspA-2*) (Fig. 8). The *STI* data in Table S4 shows the positive correlations between the different traits, and indicates that the higher *STI* values, and the stronger drought tolerance. These results revealed that transgenic *Ubi::SeCspA* lines had stronger drought tolerance than the control plants.

## Discussion

### *SeCspA* and *SeCspB* enhanced cold tolerance in *Arabidopsis*, but not in wheat

Cold shock domain (CSD) proteins in plants differ from the CSPs identified in prokaryotes. Plant CSD proteins typically contain an N-terminal CSD and a C-terminal glycine-rich region, and these regions are often interspersed with varying numbers of retroviral-like CCHC zinc finger domains^22^. Four CSD protein genes (*AtCSP1* to *AtCSP4*) were identified in *Arabidopsis*^23^. Expression of *AtCSP1, AtCSP2,* and *AtCSP3* found to be induced by cold shock treatment^24^,^25^,^26^. Overexpression of *AtCSP3* enhanced freezing tolerance in *Arabidopsis*^27^. In *E. coli*, CSPs are responsive to cold and function as RNA chaperones that are essential for bacterial growth under low temperature conditions^15^,^16^. *E. coli* CSPs share a domain with *Arabidopsis AtCSP3* and play important roles in cold tolerance^27^. Our results show that modified *E. coli CspA* and *CspB* genes enhanced resistance to cold stress when they were overexpressed in *Arabidopsis* (Fig. 2A,B), suggesting that these synthetic genes had similar functions to *Arabidopsis AtCSP3* in conferring cold tolerance in *Arabidopsis.*

At low temperatures, deletion of *CspA* is known to affect the growth of bacteria, whereas deletion of *CspB* did not significantly effect on growth^13^. Induction of *E. coli* cold shock protein CspA does not enhance transcription, but increases mRNA stability during low-temperature incubation^28^. Thus, *CspA* may be more important to cold adaptation than *CspB*. However, *SeCspA*, like *SeCspB*, did not significantly increase plant resistance to cold in transgenic wheat lines as compared to control plants (Fig. S7). It is possible that overexpression of *SeCspA* and *SeCspB* were insufficient to increase cold tolerance beyond the level already possessed by the particular winter wheat parent, which was already adapted to local conditions.

### *SeCspA* and *SeCspB* changed physiological indices and affected the expression of stress-responsive genes in transgenic plants

We measured drought-related physiological indices of transgenic *Ubi::SeCspA* and *Ubi::SeCspB* wheat lines in an attempt to determine the effects of these genes when they were overexpressed in wheat. The MDA content and the free proline content, and the rates of water loss, can be used experimentally as physiological indices of plant drought tolerance. Chlorophyll content directly affects plant photosynthesis. Under drought stress, the free proline content and the chlorophyll content of the transgenic wheat lines were higher than those of the control plants, while the MDA content and the water loss rates of the transgenic wheat lines were lower than those of the control plants (Figs 4E–G and 5C).

*E. coli* CSPs share a domain with *Arabidopsis AtCSP3*. Overexpression of *AtCSP3* in *Arabidopsis* enhanced salt and drought tolerance through up-regulated expression of stress-related proteins^29^. *SeCspA* and *SeCspB* share a domain with *Arabidopsis AtCSP3* and influenced expression of stress-related genes in the transgenic *Arabidopsis* and wheat plants (Fig. 6). Our study showed that overexpression of *SeCspA* and *SeCspB* in wheat resulted in up-regulation of *TaCDPK3*. CDPKs play important roles in stress signal transduction and can regulate the expression of salt and drought tolerance-related genes, which can be activated by ABA^30^. *RAB* plays a crucial role in plant responses to ABA^31^. However, overexpression of *SeCspA* and *SeCspB* in wheat affected the expression of ABA signaling-related genes under stress conditions, *TaRAB. TaMYB32,* a salt-inducible gene, enhanced salt stress tolerance in transgenic *Arabidopsis* overexpressing *TaMYB32*^32^. *TaWRKY2*, a WRKY-type transcription factor, is involved in multiple aspects of plant growth, development and stress response. Transgenic *Arabidopsis* plants overexpressing *TaWRKY2* exhibited improved salt and drought tolerance compared with controls^33^. Wheat ERF transcription factor members play crucial roles in regulating stress responses. Recent investigations indicated that overexpression of *TaERF3* promotes tolerance to salt and drought stresses in wheat^34^. *TaWD40*, repeat-containing WD40 proteins, acting as scaffolding molecules and promoting protein activity in protein–protein or protein–DNA interactions^35^. *TaWD40D* overexpression wheat lines enhanced tolerance to salt and drought stresses in wheat^36^. *GST*, plays an important role in resisting various stresses, is ROS-scavenging enzymes are known to be involved in redox homeostasis of cells^37^,^38^. *DHN* and *LEA* positively contributed to plant tolerance to cold, drought, and salt stresses in other plant species^39^. In this study, the transcript levels of these stress-related genes were up-regulated in the *SeCspA* and *SeCspB* overexpressing wheat lines relative to the control wheat plants. These results show that overexpression of *SeCspA* and *SeCspB* in wheat affected the expression of these stress-related genes.

Drought stress induces the accumulation of the plant hormone abscisic acid (ABA), and this compound is known to contribute to stomatal closure and thereby prevent water loss^40^. We observed that the stomata of *Ubi::SeCspA* and *Ubi::SeCspB* transgenic wheat plants closed rapidly following ABA treatment (Fig. 5A,B), which implied that the *Ubi::SeCspA* and *Ubi::SeCspB* transgenic wheat plants were more sensitive to ABA than were the control wheat plants, which maybe contribute the stomata of *Ubi::SeCspA* and *Ubi::SeCspB* transgenic wheat plants close rapidly under drought stress conditions and resulted in reduced transpiration and water loss, improving drought resistance.

### *SeCspA* improves wheat stress tolerance

Field experiments are essential to verify experimental differences that are observed in greenhouse experiments^10^. We performed field tests on our transgenic *Ubi::SeCspA* and *Ubi::SeCspB* wheat plants. Wheat was sown in early October and harvested in mid-June of the following year. Rainfall in Shijiazhuang is mainly concentrated in mid-June to August (Fig. S8). During the whole growing season of wheat, the Shijiazhuang area is in a period of low rainfall. Rainfall in Shijiazhuang at this time does not meet the water requirement for wheat growth; this site is therefore suitable for drought response trials with transgenic wheat (Fig. S8). The soil water content of the experimental plots was monitored during the whole wheat growing season. Under the well-irrigated conditions, the soil water content of the experimental plot area was about 18.5% to 26.9% (average of two years) during the entire growth cycle (Table S5), which suggested there should have enough water for wheat to grow. Under the water-limited conditions, the soil water content of the experimental plot area was about 8.7% to 12.1% (average of two years) during the late growth stage (Table S5), which suggested that wheat grown in this area would experience drought conditions.

Different parameters have been used to evaluate the drought resistance of crops in the field. For example, growth and senescence of the maize canopy was evaluated using normalized differences in vegetation indices under drought stress^41^. Seedling leaf senescence scores were used as an index for evaluating drought resistance in rice^42^. We used *SSI, STI,* and *TOL* to evaluate drought resistance of our transgenic wheat plants. *SSI*, in particular, can represent the drought resistance of the transgenic wheat. Cluster analysis between *SSI* and grain yield showed that group I (Control) performed poorly in all environments and could not acclimatize to the changing environment, the *Ubi::SeCspB* transgenic wheat lines in group II performed well only under normal irrigated conditions, indicating that *Ubi::SeCspB* transgenic wheat lines would not be suitable for cultivation in arid regions (Fig. 8). Group IV members performed best in all environments (Fig. 8), suggesting that *Ubi::SeCspA* transgenic wheat lines have relatively less drought sensitivity than the control genotype under drought conditions.

## Materials and Methods

### Modification of the *E. coli CspA* and *CspB* genes

In order to improve the expression of exogenous *E. coli CspA* and *CspB* in plant cells, nucleotide changes were made to the DNA sequence in order to increase the overall G+C content; the purpose behind these changes was to increase the number plant-preferred codons without changing the amino acid sequence of the CspA and CspB proteins^43^. The modified sequences were named *SeCspA* and *SeCspB* (Fig. S1).

### Generation of transgenic *Arabidopsis* and wheat plants

*SeCspA* and *SeCspB,* with *Sma*I and *Spe*I enzyme sites, were recombined into the pBI121 vector (kanamycin) under control of the cauliflower mosaic virus (CaMV) 35 S promoter, resulting in 35 S::*SeCspA* and 35 S::*SeCspB* constructs. The recombinant constructs were introduced into *Agrobacterium tumefaciens* strain EHA105, which was then used for the transformation of plant by the floral dip method. *Arabidopsis (Arabidopsis thaliana*) ecotype Columbia (Col-0) were grown until flowering under normal growth conditions. The recombinant strains carrying recombinant vector *SeCSP*-pBI121 were then used to transform *Arabidopsis*. Homozygous T3 lines were obtained and used for further experiments. The methods were described by Clough and Bent, 1998^44^. The leaves of three-week-old T3 transgenic *Arabidopsis* lines were taken and used for extracting total RNA (RNAprep plant kit, TIANGEN), and then reverse transcribed into cDNA using a PrimeScript First-Strand cDNA Synthesis kit (Takara). Transcript levels of *SeCspA* and *SeCspB* in transgenic *Arabidopsis* lines were detected by semi-quantitative PCR.

For generation of the transgenic wheat expression vectors, *SeCspA* and *SeCspB* were recombined into the pAHC25 vector^45^ under control of the ubiquitin (Ubi) promoter, and *bar* was used as the plant selection marker gene (Fig. S5A). Calli of immature embryos of common wheat cultivar KN199 (winter wheat) were cultured for one week at 26 °C in darkness and used in the transformation experiments. Bombardment was performed as described by Xu *et al*.^46^. After bombardment, calli were cultured and incubated on 1/2 MS medium containing 3 mg/L Bialaphos for 4 weeks (Fig. S5B). Regenerated plantlets were transferred to plantlet strengthening medium to allow vigorous root development following the methods described in Pellegrineschi *et al*.^47^, (Fig. S5C). Rooted plants were potted into soil and grown to maturity in a greenhouse. The transgenic wheat lines were generated by particle bombardment and propagated for two generations in the greenhouse.

Genomic DNA was extracted from the transgenic wheat lines and the control wheat plant tissue samples for PCR analysis using the CTAB method^48^. All T1 seeds from the T0 parents were planted in the field and allowed to set T2 seeds. When T1 wheat seedlings had grown into three-leaf plantlets, the genomic DNA from the T1 seedlings was extracted and analyzed by PCR analysis using the specific primers detailed in Table S6 (UBI-F from the vector, SeCspA-R, SeCspB-R). All PCR-positive T2 wheat seeds from unique (single) T1 wheat plants were harvested together and then planted in a field to allow plants to set T3 seeds. One of the homozygous T3 wheat plants from each PCR analysis-positive T2 wheat line was randomly examined via for Southern blot analysis; Southern blot-positive transgenic wheat plants were allowed to produce T4 seeds (Fig. S6). Four homozygous T4 transgenic wheat line progenies from each Southern blot-positive T3 transgenic wheat lines were harvested together and used for subsequent biochemical analyses.

### Performance of transgenic *Arabidopsis* under stress treatment

One hundred homozygous T3 seeds of the *35 S::SeCspA* and *35 S::SeCspB* transgenic lines, and control plants, were sown on half-strength MS medium [1/2 strength Murashige and Skoog medium with salt and vitamins (PhytoTechnology Laboratories) further supplemented with 0.8% (w/v) agar, 30 mg L^–1^ hygromycin B (Sigma-Aldrich), and 2% (w/v) sucrose, pH 5.7] containing different concentrations of NaCl (0, 50, 100, and 150 mM) or PEG 6000 (0, 4% and 8%) and stored in a refrigerator (4 °C) and vernalized for 3 days before transfer to a controlled environment chamber (16 h light/8 h darkness, 23 °C, 70% relative humidity) for growth. After 4 days, germination rates were scored prescribed days, essentially as described by Kim *et al*.^49^,^50^.

For seedling phenotype analysis of the control and the *35 S::SeCspA* and *35 S::SeCspB* lines under salt treatment, 9-day-old seedlings of transgenic lines and control plants (including three-day vernalization treatment) were transferred to half-strength MS (Murashige and Skoog) agar plates supplemented with 100 mM NaCl and grown for one week under long-day conditions (16 h light/8 h dark) at 23 °C with 70% relative humidity, and the fresh weights of the aerial parts and the primary root lengths of the plants were measured after one week of treatment^51^.

For the drought treatment, three-week-old plants grown in soil (including three-day vernalization treatment) under normal conditions (16 h light/8 h dark, 23 °C, 70% relative humidity) were exposed to drought conditions for one week (plants were not watered), and the plants were then re-watered for growth recovery for one week. Similarly, for cold tests, three-week-old seedlings were transferred to −5 °C for 12 h and then returned to normal growing conditions for recovery for one week. Survival rates were recorded after recovery for one week. The experiments were repeated for several times and the results were consistent. One set of the experiments was shown.

### Effect of salt, drought and cold stress on the growth of wheat

Wheat seeds were sown in a 96-well plates that were level with the surface of a hydroponic solution and grown in a controlled environment chamber under normal conditions (23 °C, 16 h light/8 h darkness) for one week. The seedlings were then treated with salt stress (200 mM NaCl in the hydroponic solution). After one week of salt treatment, the phenotypes of transgenic and control wheat plants were observed and the relative Na^+^ content in the leaves were measured using an atomic absorption spectrometer. The fresh weights of the transgenic and control plants were measured with MS Analytical Balances (Mettler Toledo).

The effects of drought on physiological indices were evaluated for two-week-old seedlings. Seven-day-old plants were treated with drought conditions for one week before measuring physiological indices. All of the measurements were repeated three times. MDA content was assayed according to the method reported by Lv *et al*.^52^. Wheat leaf samples (0.1 g) were used, and absorbance values at 450, 532, and 600 nm were measured with a spectrophotometer (Perkin-Elmer Lambda 25, Boston, MA, USA). The MDA content was calculated using the following formula: C (1 mol/L) = 6.45 (OD532–OD600)–0.56 OD450. About 0.1 g of wheat leaf was used for the measuring the proline content; the samples were treated with 3% (w/v) sulphosalicylic acid and were boiled for 1 h. The proline content was measured with ninhydrin, which was measured at 520 nm, and a proline standard liquid was used as a reference for generation of a standard curve^53^. Chlorophyll content was measured with a traditional spectrophotometer method; pigments were extracted overnight in 90% ethanol^54^.

Seven-day-old transgenic and control wheat seedlings were exposed to low temperature (−4 °C) for one week in a controlled environment chamber (16 h light/8 h darkness). The phenotypes were then observed and the root lengths were measured.

### Relative electric conductivity

The sample leaves (0.1 g, wheat seedlings in the two leaf stage) of transgenic wheat lines and control plants seedlings (with natural growth condition and with 2 h of −8 °C cold treatment) were put into 20 ml of distilled water and a vacuum (test tube) was applied for 30 min, and then surged for 2 h to measure the initial electric conductance (S1) (25 °C). A test tube was filled with leaf discs and distilled water, the mixture was cooked (100 °C) for 30 min and then reduced to room temperature (25 °C) to determine the final electric conductance (S2). The relative electric conductivity was evaluated as: REC = S1 × 100/S2.

### Stomatal aperture and water loss analysis

Leaves of 7-day-old wheat plants were collected and incubated for 4 h in stomatal-opening solution (0.05 M KNO_3_/10 mM, MES/50 μM CaCl_2_, pH = 6.15) under high light conditions (810 μmol•m^−2^•s^−1^, 25 °C). The leaves were then transferred into an ABA-containing solution (0, 15 μM, and 20 μM) for 2 h. The adaxial sides of the leaf epidermis was peeled off using a cutter blade and leaves were then mounted on slides and observed with a confocal laser scanning microscope (CLSM).

To measure water loss, leaves of 7-day wheat plants were excised and placed on a bench (25 °C, 30% relative humidity), and the initial fresh weights of plants was measured at 0 h and recorded W0, and then after 0.5 h, 2 h, 3 h and 5 h, the fresh weights of plants was measured and recorded Wn (n = 0.5, 2, 3, 5), respectively. The water loss rate was evaluated as: (W0 – Wn)*100/W0.

### *De novo* sequencing of the control and transgenic wheat plants

Triplicate samples (one-week-old wheat seedlings, the two-leaf stage wheat seedlings), comprising three independent plants of each line, were used for RNA sequencing experiment. Total RNA was extracted from control wheat plants and the transgenic wheat lines using TRIzol reagent (Invitrogen) and then treated with RNase-free DNaseI to remove contaminating DNA. The quality and quantity of RNA were examined using an Agilent 2100 Bioanalyzer (Agilent Technologies) using the procedures described in Duan *et al*.^55^. RNA sequencing was performed on a HiSeq 2000 instrument (Illumina).

We used pair-end reads to assemble the hexaploid wheat transcriptome. Three state-of-the-art assemblers were used: Trinity was employed with the ALLPATHSLG error correction^56^; ABySS version 1.2.5 with the scaffolding option off and contig end erosion off was carried out for individual k-mer assemblies^57^; and trans-ABySS version 1.2.0 was used to merge the individual k-mer assemblies with default parameters^58^. The *de novo* assembly process was divided into the following four steps: pre-assembly, merging different samples, removal of redundancy, and scaffolding. Every detail of these steps and how these steps influenced assembly performance are reported in Duan *et al*.^55^. After optimization, the assembled transcripts were compared to the Sangerderived ESTs in terms of both continuity and accuracy. The differentially expressed genes were estimated by using DEseq package and considered to be significant at p-value < 0.05 and absolute fold-change ≥2- fold. Gene ontology analysis was performed by Blast2go software^59^.

### QRT-PCR

Wheat seedlings at the two leaf stage that were grown under normal condition were sampled and frozen in liquid nitrogen for 10 min, and then transferred into −80 °C refrigerator for saving. Total RNA was extracted from wheat leaves using an RNAprep plant kit (TIANGEN) and reverse transcribed into cDNA using a PrimeScript First-Strand cDNA Synthesis kit (Takara). SYBR master mix (TIANGEN) was used for qRT-PCR. qRT-PCR was performed following the methods described in Liu *et al*.^60^ using an ABI Prism 7500 real-time PCR system (Lifetech). Wheat *actin*, as an internal reference, was used to normalize all data. Three biological replications for each line were performed in each test. The relative transcript levels of stress-responsive genes (*TaMYB32, TaWRKY2, TaWD40, TaCDPK3, TaGST, TaRAB18, TaLEA, TaDHN* and *TaERF3*) were calculated using the 2^−ΔΔCT^ method^61^. All primers for the stress-responsive genes are listed in Table S6.

### Response of transgenic wheat to drought stress in the field

Field trials for the drought response of the transgenic wheat lines were conducted at Shijiazhuang in Hebei province, China. The soil type was a loam. Soil properties at the 0–40 cm depth were pH 7.5–8.5, 1–2.2% organic matter, 30–99 ppm of inorganic N, 3–20 ppm of available Olsen P, and 100–200 ppm of K. The trace elements iron, copper, and manganese were abundant (4.5–10 ppm Fe, 1–2 ppm Cu, 10–15 ppm Mn), the soil bulk density was 1.301 g cm^3^, the saturation point was 43%, and the wilting coefficient was 6–7%. During the 2013 and 2016 growing season, the mean monthly temperatures in Shijiazhuang varied from 3 °C (January) to 28 °C (June), and Shijiazhuang received 159.4 and 120.1 mm of rainfall from January to June (Fig. S8).

Drought response tests were conducted from 2013 to 2014 and 2015 to 2016 with T5 and T7 generation plants. T5 and T7 generation control wheat plants and the transgenic wheat lines were grown during the 2013 to 2014 and 2015 to 2016 growing season on an experimental farm near Shijiazhuang in Hebei province, China. The experimental design was a two factor split-plot design with three replications (separate plots at the same site), water treatments were applied to entire main plots; genotypes were included as subplots. Each genotype was planted in 6 m^2^ plots with 4 m length, 1.5 m wide and seeds were sown by a bedder planter with an interval of 5 cm.

The field was irrigated before sowing in order to saturate the soil profile and to facilitate germination. T5 and T7 generation control wheat plants and the transgenic wheat lines were planted on October 2, 2013 and October 5, 2015 (average soil water content of 0–40 cm was 20.01%). The well-watered treatment plots were given a total of 240 mm irrigation (across four applications) at the over-wintering stage (September 19–20, 2013 and 2015), the stem elongation stage (March 3–5, 2014 and 2016), the flowering stage (April 25–30, 2014 and 2016), and the grain filling stage (May 21–25, 2014 and 2016). While the water-stress treatment was given a total of 90 mm irrigation (two applications) at the wintering stage (September 19–20, 2013 and 2015) and the stem elongation stage (March 3–5, 2014 and 2016). The average soil water content of 0–40 cm at the flowering stage (April 25–30, 2014 and 2016) was 10.3% (Water Stress) and 24.5% (Well-Watering), respectively. The average soil water content of 0–40 cm at the grain filling stage (May 21–25, 2014 and 2016) was 9.2% (Water Stress) and 24% (Well-Watering), respectively. The major agronomic traits of the transgenic and control wheat lines were observed for 20 plants from each of five sampling points for each plot. Drought resistance indices of the transgenic and control wheat lines were calculated using the following relationships: (1) *SSI* = [1 − (*Ys*)/(*Yp*)]/[1 − (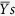)/(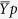)]^62^, where *Ys* and *Yp* are the indices of the transgenic and control wheat lines evaluated under stress and non-stress conditions, and 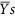 and 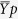 are the mean yields over all of the transgenic and control wheat lines evaluated under stress and non-stress conditions; (2) *TOL* = *Yp* − *Ys*^63^; (3) *STI* = (*Yp* − *Ys*)/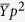 64. The soil samples of each plot were weighed as fresh weights (FW) and then dried in an electric drying oven at 80 °C for 48 h and weighed as dry weights (DW). The soil water content (%) was calculated using the following formula: [(FW − DW)/DW] * 100.

## Additional Information

**How to cite this article:** Yu, T.-F. *et al*. Improved drought tolerance in wheat plants overexpressing a synthetic bacterial cold shock protein gene *SeCspA. Sci. Rep.* **7**, 44050; doi: 10.1038/srep44050 (2017).

**Publisher's note:** Springer Nature remains neutral with regard to jurisdictional claims in published maps and institutional affiliations.

## Supplementary Material

Supplementary Data

## Figures and Tables

**Table 1:** Stress susceptibility indices (*SSI*), Stress tolerance (*TOL*), and Stress tolerance index (*STI*) for yield traits of the transgenic lines (average of two years).

Indices	SSI			TOL			STI		
Agronomic trait	1000-grain weight	Panicle number	Grain yield	1000-grain weight	Panicle number	Grain yield	1000-grain weight	Panicle number	Grain yield
*SeCspA-1*	0.272^a^	0.746^a^	0.864^a^	0.815^a^	0.2	1.441^a^	1.057^a^	1.031^a^	0.610^a^
*SeCspA-2*	0.317^a^	0.756^a^	0.872^a^	0.940^a^	0.2	1.448^a^	1.048^a^	1.005^a^	0.601^a^
*SeCspB-1*	1.468	1.159	1.094	4.130	0.3	1.809	0.852	0.948	0.491
*SeCspB-2*	1.535	1.167	1.097	4.310	0.3	1.810	0.841	0.935	0.487
control	1.514	1.193	1.109	4.180	0.3	1.817	0.813	0.893	0.474

^a^Indicates significant (P < 0.05) improvement relative to nontransgenic control.
