# Supplementary material for: Improved drought tolerance in wheat plants overexpressing a synthetic bacterial cold shock protein gene SeCspA
Source: Sci Rep. 2017 Mar 10;7:44050. doi: 10.1038/srep44050 (PMC5345034; doi:10.1038/srep44050)
Supplement: Supplementary Data [file srep44050-s1.doc]

**Supporting information**

**Improved drought tolerance in wheat plants overexpressing a synthetic bacterial cold shock protein gene *SeCspA***

Tai-Fei Yu^1,†^, Zhao-Shi Xu^1,†,*^, Jin-Kao Guo^2^, Yan-Xia Wang^2^, Brian Abernathy^3^, Jin-Dong Fu^1^, Xiao Chen^1^, Yong-Bin Zhou^1^, Ming Chen^1^, Xing-Guo Ye^1^ and You-Zhi Ma^1,*^

1 Institute of Crop Science, Chinese Academy of Agricultural Sciences (CAAS)/National Key Facility for Crop Gene Resources and Genetic Improvement, Key Laboratory of Biology and Genetic Improvement of Triticeae Crops, Ministry of Agriculture, Beijing 100081, China

2 Shijiazhuang Academy of Agricultural and Forestry Sciences, Research Center of Wheat Engineering Technology of Hebei, Shijiazhuang, Hebei 050041, China

3 Center for Applied Genetic Technologies, Department of Plant Sciences, University of Georgia, 30602, Athens, GA, United States

† Authors contributed equally to the present work.

* Correspondence (fax: +86 010 8210 8789; e-mail: xuzhaoshi@caas.cn (Z-S X) or mayouzhi@caas.cn (Y-Z M))

Supplementary Information contains:

Supplementary Tables S1-S6

Supplementary Figures S1-S8

Additional supporting information can be found in the online version of this article:

**Table S1** Annotation of genes up-regulated in transgenic wheat plants.

| Gene | Annotation | Fold change ( OE/WT) |
| --- | --- | --- |
| **Transcription factor (Gene ID)** |  |  |
| Traes_2BL_0AED4E377 | NAC domain transcription factor | 3.6339 |
| Traes_3DL_21E2888C9 | Myb-domain transcription factor | 3.5355 |
| Traes_1AL_A16202A31 | ethylene responsive transcription factor 6 | 3.4413 |
| Traes_5DL_0DFA1AAF7 | AP2-like ethylene-responsive transcription factor | 3.3343 |
| Traes_1BS_824DD258F | Zinc finger protein-transcription factor | 3.0933 |
| Traes_6DL_569FCEEC5 | Ethylene-responsive transcription factor ERF012 | 3.2778 |
| Traes_3DS_23D92FFEE | transcription factor IIIB | 3.0933 |
| Traes_3DS_695C4DC64 | transcription factor HBP-1b | 3.0933 |
| Traes_5AL_89A09BC1B | Transcription factor bHLH92 | 3.0933 |
| Traes_6AL_A8903FD22 | AP2 domain CBF transcription factor | 3.0031 |
| Traes_5DL_91AE6CA271 | CRT/DRE binding transcription factor | 2.9007 |
| Traes_7AL_C3FEBECBC | WRKY39 transcription factor | 2.8563 |
| Traes_5AS_EBCEA6601 | Transcription factor PIF3 | 2.0378 |
| **Kinase (Gene ID)** |  |  |
| Traes_3DL_B6EAFCDDA | LRR receptor-like serine/threonine-protein kinase | 4.3343 |
| Traes_7AL_85123FD23 | Cysteine-rich receptor-like protein kinase 10 | 4.2189 |
| Traes_3DS_5D2EB4995 | Protein kinase APK1B, chloroplastic | 3.7214 |
| Traes_5DL_0DFA1AAF7 | Putative serine/threonine-protein kinase | 3.3343 |
| Traes_7AL_07AFCB20D | Putative serine/threonine-protein kinase NAK | 3.3343 |
| Traes_2DS_D690D8166 | CBL-interacting protein kinase 29 | 2.9558 |
| Traes_3DL_67169C792 | Leucine-rich repeat receptor protein kinase EXS | 2.8038 |
| Traes_6BS_814D20B55 | Calcium-dependent protein kinase | 2.1629 |
| Traes_6BS_2996C7AD8 | Serine/threonine protein kinase Stpk-B | 2.0376 |
| **Function protein (Gene ID)** |  |  |
| Traes_3B_4F509404E | Dehydrin (DHN) | 11.7047 |
| Traes_2BL_2A98E7B29 | Rab family | 9.2378 |
| Traes_2AL_0245451A4 | Glutathione S-transferase | 3.5974 |
| Traes_2BS_E7D211A51 | Late embryogenesis abundant (LEA) hydroxyproline-rich glycoprotein family | 2.9558 |
| Traes_5DL_89CF7F5DE | Heat shock protein 90 | 2.0381 |
| **Carbohydrate metabolism-related proteins (Gene ID)** |  |  |
| Traes_4AL_F68BCA960 | UDP-glycosyltransferase 85A2 | 2.0489 |
| Traes_2DL_3D4292439 | UDP-N-acetyl glucosamine transferase subunit ALG13-like protein | 3.6339 |
| Traes_3AL_7160E6873 | Alpha-amylase | 3.4413 |
| **Oxidation-reduction process (Gene ID)** |  |  |
| Traes_5DL_A00D602EC | cytochrome P450 94C1-like | 3.3176 |
| Traes_5AL_221649956 | Respiratory burst oxidase-B-like protein | 3.0933 |
| **Energy (Gene ID)** |  |  |
| Traes_2AS_CF67B6097 | Isocitrate lyase | 4.8038 |
| Traes_5BL_5D43B8D06 | fructose-6-phosphate-2-kinase/fructose-2,6-bisphosphatase | 3.6339 |
| Traes_2DL_676AA4883 | Farnesyl pyrophosphate synthetase | 2.9915 |
| Traes_7BL_DF779F2AE | Beta-glucosidase 34 | 2.9558 |
| Traes_5DS_900AE89AC | 5-methylthioadenosine/S-adenosylhomocysteine deaminase-like | 2.112 |
| Traes_4DL_26ECC8DDC | Plasma membrane ATPase | 2.8038 |

**Table S2** Plant height and heading time of control and transgenic lines under normal condition

| Line | Year | Plant height (cm) | heading time |
| --- | --- | --- | --- |
| *SeCspA-1* | 2013 to 2014 | 77.1±2.1 | Apr. 26 |
|  | 2015 to 2016 | 76.7±2.4 | Apr. 25 |
| *SeCspA-2* | 2013 to 2014 | 76.4±2.1 | Apr. 26 |
|  | 2015 to 2016 | 76.4±2.5 | Apr. 25 |
| *SeCspB-1* | 2013 to 2014 | 77.0±2.6 | Apr. 26 |
|  | 2015 to 2016 | 76.1±1.9 | Apr. 25 |
| *SeCspB-2* | 2013 to 2014 | 76.2±2.3 | Apr. 26 |
|  | 2015 to 2016 | 75.9±1.7 | Apr. 25 |
| KN199 | 2013 to 2014 | 76.9±2.3 | Apr. 26 |
|  | 2015 to 2016 | 76.0±2.1 | Apr. 25 |

**Table S3** Plant height and heading time of control and transgenic lines under drought condition

| Line | | Year | Plant height (cm) | heading time |
| --- | --- | --- | --- | --- |
| *SeCspA-1* | | 2013 to 2014 | 66.10±3.25 | Apr. 22 |
|  |  | 2015 to 2016 | 65.30±2.02 | Apr. 20 |
| *SeCspA-2* | | 2013 to 2014 | 66.90±2.15 | Apr. 22 |
|  |  | 2015 to 2016 | 64.80±2.37 | Apr. 20 |
| *SeCspB-1* | | 2013 to 2014 | 66.10±2.66 | Apr. 22 |
|  |  | 2015 to 2016 | 64.70±2.31 | Apr. 22 |
| *SeCspB-2* | | 2013 to 2014 | 65.90±2.11 | Apr. 22 |
|  |  | 2015 to 2016 | 64.10±2.36 | Apr. 20 |
| KN199 | | 2013 to 2014 | 66.70±3.17 | Apr. 22 |
|  |  | 2015 to 2016 | 64.20±2.61 | Apr. 20 |

**Table S4** Correlation coefficients between different traits and SSI, TOL, STI (average of two years).

| Trait | Drought stress | | | Non drought stress | | |
| --- | --- | --- | --- | --- | --- | --- |
| Year | SSI | TOL | STI | SSI | TOL | STI |
| Grain yield | -0.9995 | -0.9976 | 0.9996 | -0.8056 | -0.7828 | 0.8408 |
| Panicle number | -0.9423 | -0.9210 | 0.9987 | -0.8903 | -0.8623 | 0.9966 |
| 1000-grain weight | -0.9970 | -0.9954 | 0.9995 | -0.9759 | -0.9717 | 0.9938 |

**Table S5** Soil water content during the growing season (average of two years).

|  | Soil water content (%) | | | |
| --- | --- | --- | --- | --- |
| Stages | No water | | Well-watered | |
|  | 0-20 cm | 20-40 cm | 0-20 cm | 20-40 cm |
| over-wintering | 18.8±2.3 | 21.3±2.5 | 18.5±2.4 | 21.9±2.1 |
| stem elongation | 11.3±1.1 | 12.1±1.1 | 20.2±2.1 | 26.6±1.5 |
| flowering | 9.5±1.3 | 11.0±0.9 | 21.9±1.6 | 27.1±2.4 |
| grain filling | 8.7±0.9 | 9.7±1.4 | 21.1±1.7 | 26.9±1.7 |

**Table S6** The specific primers of related genes in this article

| Primers | Sequences |
| --- | --- |
| TaCDPK3-F | AGAAAGGCATCACGGCACTACTC |
| TaCDPK3-R | CATCAGTGGTTCAGAAAAGCAGG |
| TaLEA-F | GAGCAATACTAGCAGTGAGATTTAC |
| TaLEA-R | GTACTGTAGAAGGCTCGTGAAC |
| TaWD40-F | CGGTGTGGCTTCGGCA |
| TaWD40-R | GCTTCGTGGCGTCGCT |
| TaGST-F | CATTTTGCTGGCAGTTTA |
| TaGST-R | TGTTCGGTTGCTTGATGTAGT |
| TaDHN-F | TGGGACGGGCTCAGTGCT |
| TaDHN-R | ATGGGCGGGAGGAGGAAG |
| TaRAB-F | GCATTTCCAGCCCACGAG |
| TaRAB-R | CGGTCTGTTGCTGGTCGC |
| TaWRKY-F | TCGATCGCCATGTCCTCCTC |
| TaWRKY-R | AGCGACTCGACGAACATGTCG |
| TaERF3-F | CTCGGCGGTGTCGTAGGTGC |
| TaERF3-R | AGCAATCAGGCAAAGCAACC |
| TaMYB32-F | TGAGATGGACTTCTGGGTCAC |
| TaMYB32-R | GGTATTATCAGGCTTTCAGTCC |
| Wheat actin-F | CTCCCTCACAACAACCGC |
| Wheat actin-R | TACCAGGAACTTCCATACCAAC |
| UBI-F | ATGCTCATCCTGTTGTTTGGTG |
| SeCspA-R | CTTGTAGCCGTCGTTTTGGATG |
| SeCspB-R | AAGCCCTCGCCTTGGATGG |

**Figure S1**


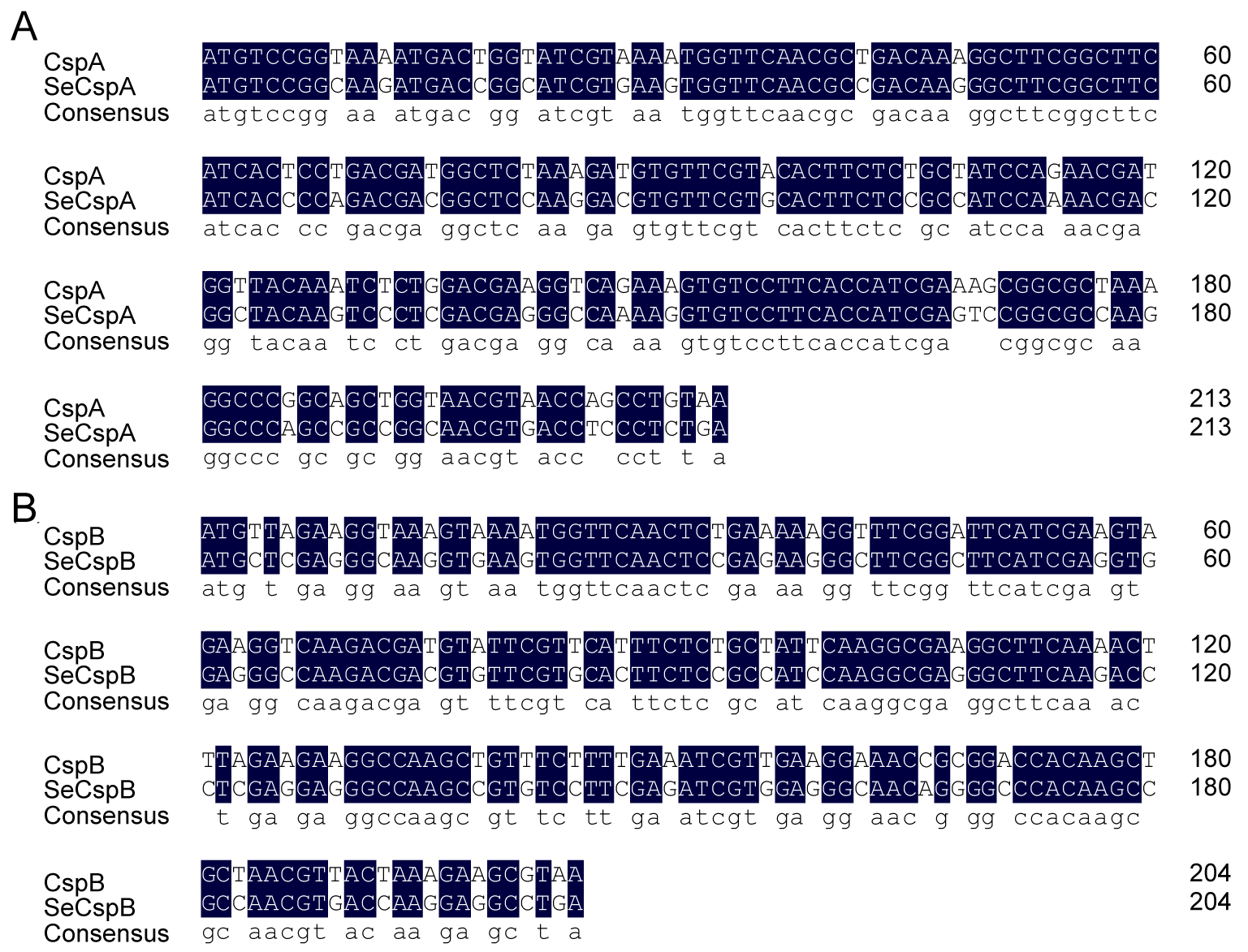


**Figure S1** Modified sequence of *E. coli* cold shock protein genes. (A) Modified sequence of the *E. coli* *CspA* gene. (B) Modified sequence of the *E. coli* *CspB* gene.

**Figure S2**


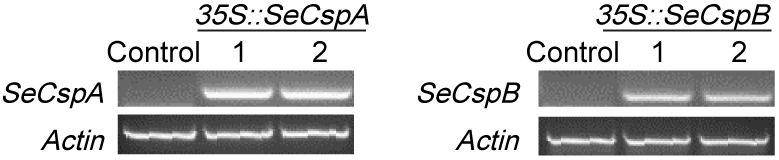


**Fig. S2** Transcript levels of SeCspA and SeCspB in transgenic *Arabidopsis* lines.

**Figure S3**


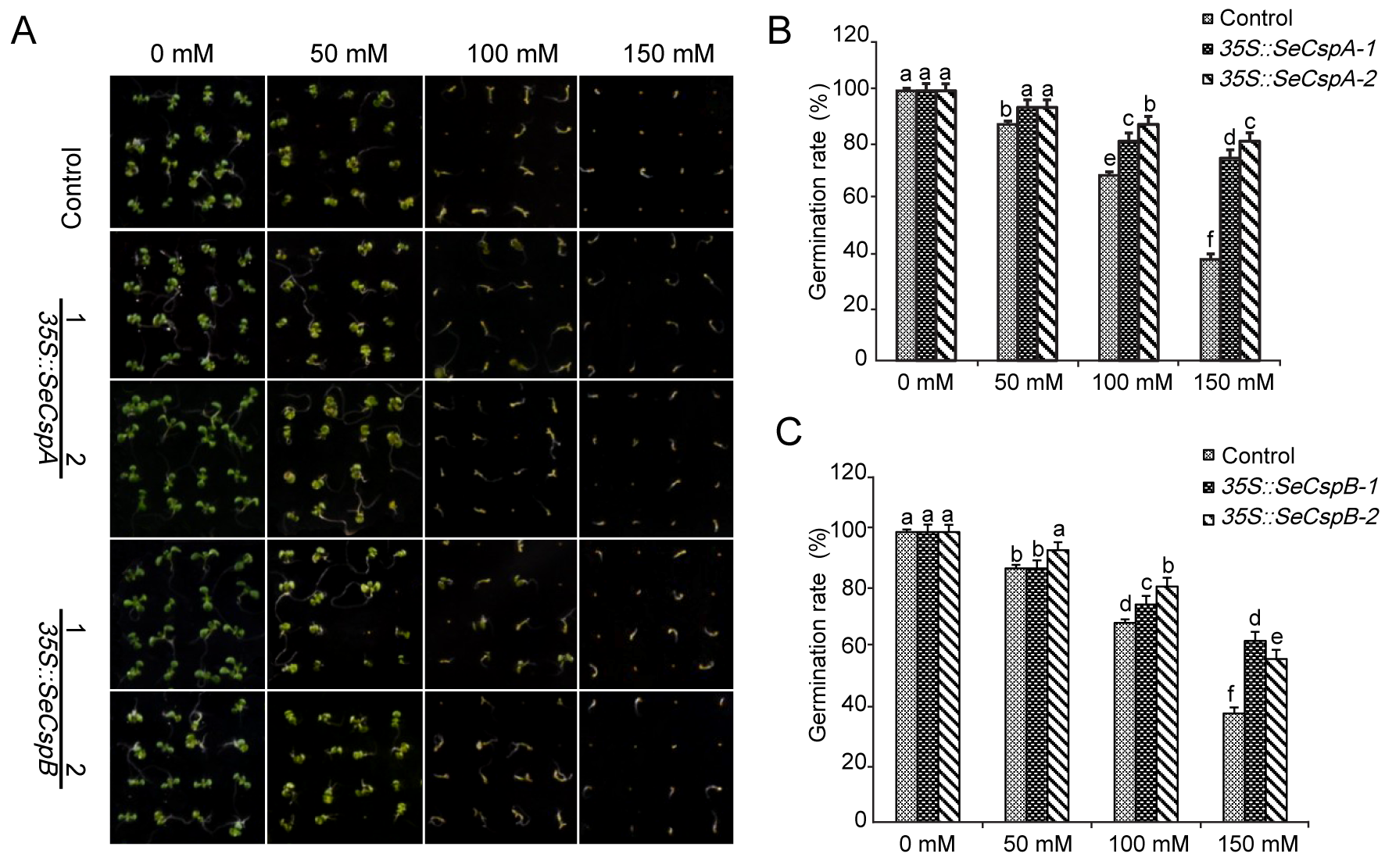


**Figure S3** Effect of salt stress on the germination of transgenic and control *Arabidopsis* seeds. (A) Phenotypes of germinated seedlings after sowing on MS medium containing NaCl. (B) and (C) Germination rates were scored after sowing. Vertical bars bearing different letters in (B) and (C) indicate significant differences at P < 0.05 and error bars represent standard errors.

**Figure S4**


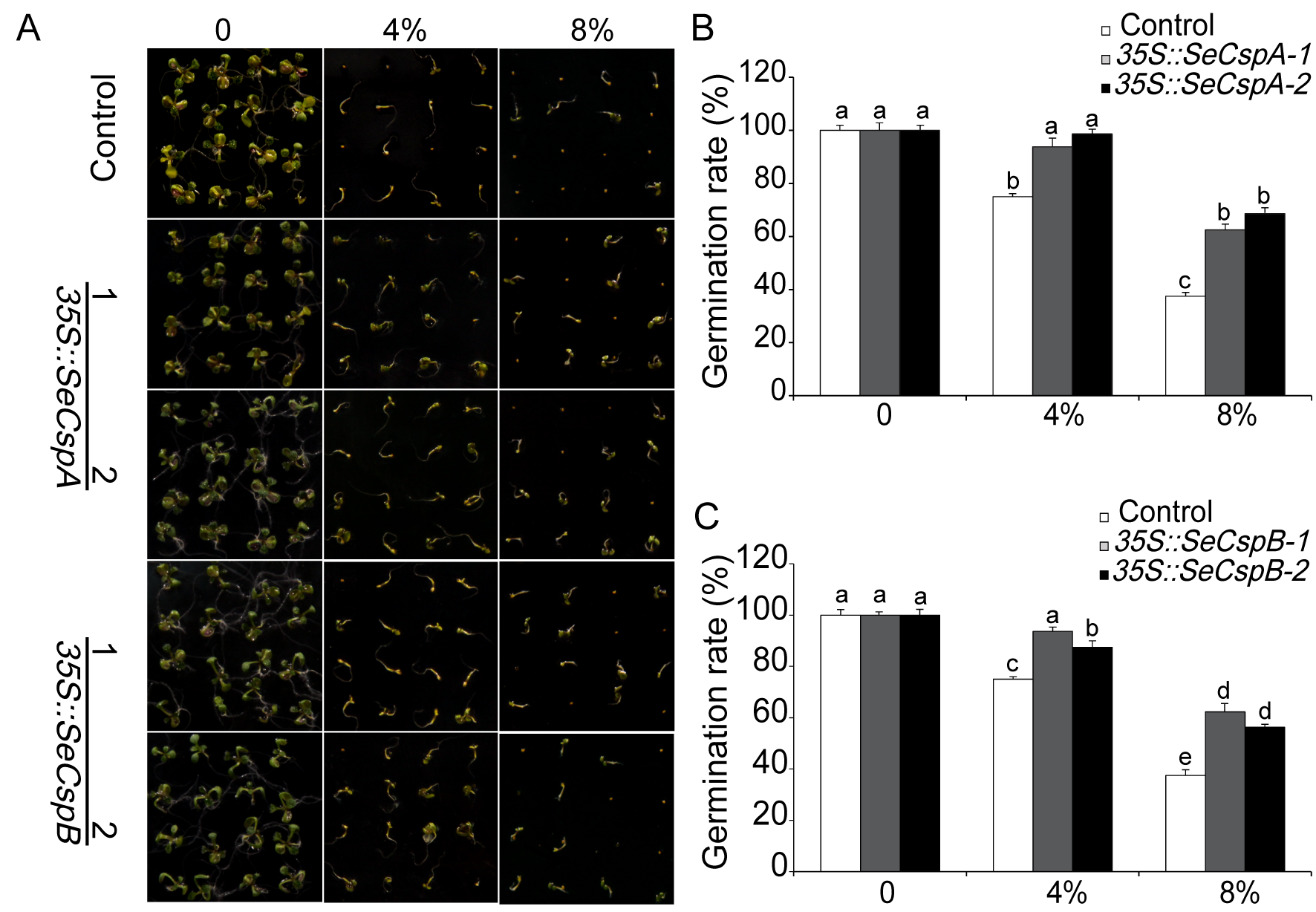


**Figure S4** Effect of drought stress on seed germination of transgenic and control *Arabidopsis* plants. (A) Phenotypes of germinating seeds after sowing on MS medium containing PEG. (B) and (C) Germination rates. Germination rates were scored after sowing. Vertical bars bearing different letters in (B) and (C) indicate significant differences at P < 0.05 and error bars represent standard errors.

**Figure S5**


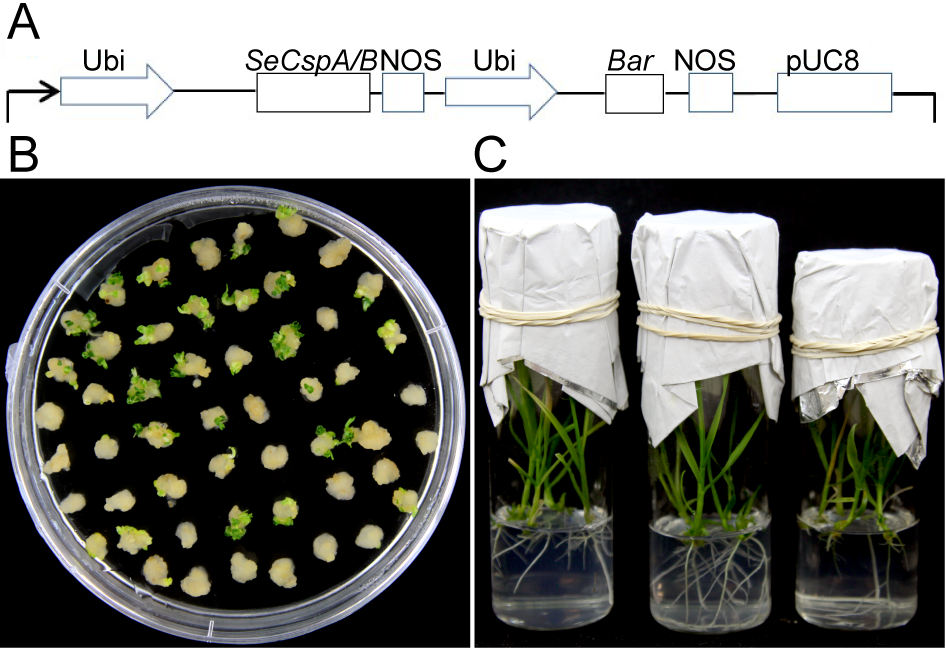


**Figure S5** Expression vector generation and genetic transformation of wheat. (A) The expression vector, plasmid pAHC25-Ubi::*SeCspA/B*-Ubi::Bar, (Ubi-*SeCspA/B*-Ubi-*Bar*). Ubi, maize ubiquitin promoter; Bar, plant selection marker gene. (B) Calli cultured on 1/2 MS medium containing 3 mg/l Bialaphos were photographed after differentiation of visible buds. (C) Regenerated plantlets grown on plantlet strengthening media.

**Figure S6**


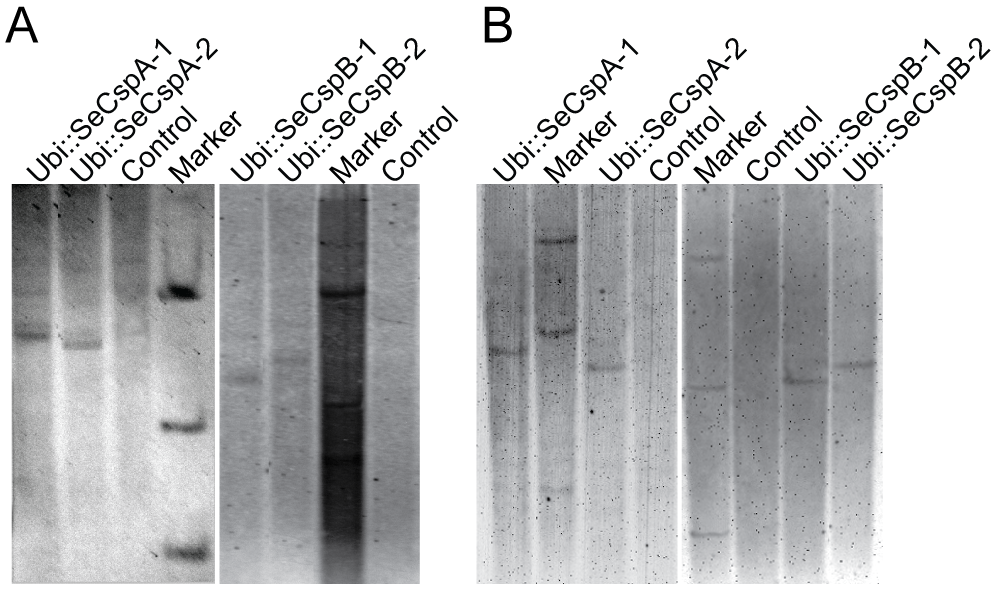


**Figure S6** Southern blot analysis of transgenic wheat lines. (A) Wheat genomic DNA digested with *Hind*III. (B) Wheat genomic DNA digested with *EcoR*I.

**Figure S7**

**
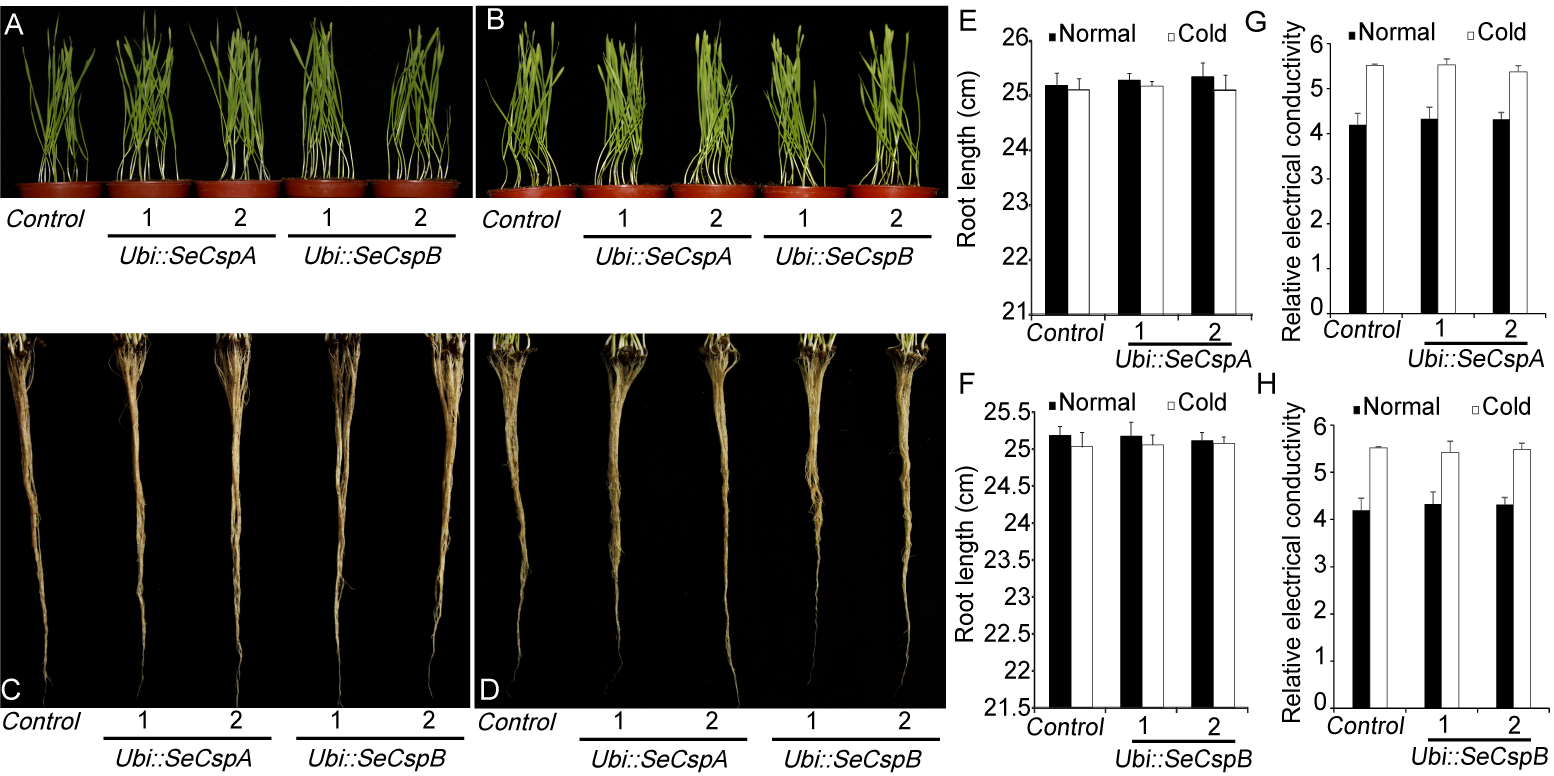
**

**Figure S7** Response of transgenic wheat lines and the control wheat plants to cold stress. (A) and (C) Phenotypes of transgenic wheat lines grown under normal conditions for two weeks. (B) and (D) Phenotypes of transgenic lines subjected to cold (-4 °C) stress. (E) and (F) Comparison of root lengths. (G) and (H) Comparison of the relative electric conductivity.

**Figure S8**


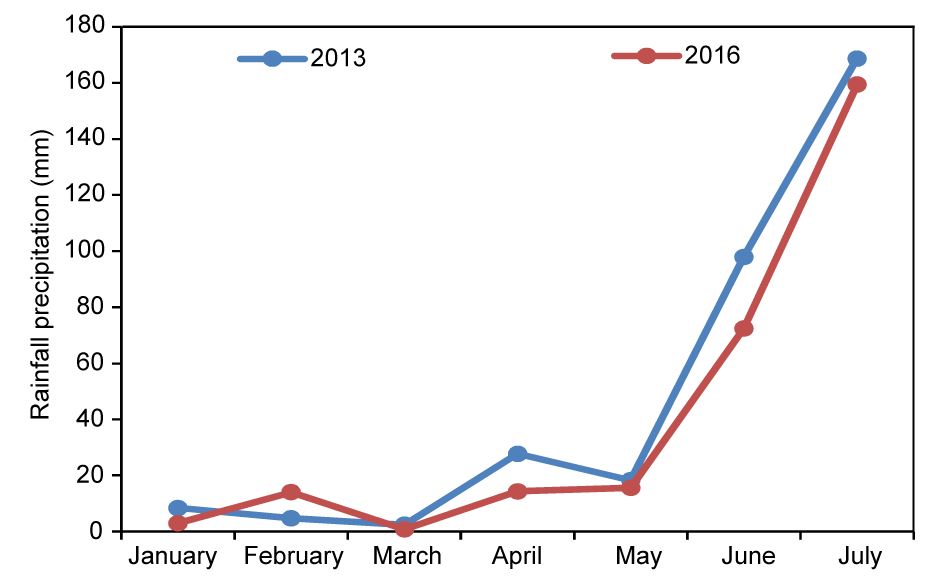


**Figure S8** Rainfall in Shijiazhuang from January to July (2013 and 2016).
